# Władysław Sterling (1877–1943)

**DOI:** 10.1007/s00415-018-8944-2

**Published:** 2018-06-20

**Authors:** Katarzyna Pekacka-Falkowska, Anna Maria Pekacka

**Affiliations:** 1 Department of History and Philosophy of Medical Sciences, Poznan University of Medical Sciences, Przybyszewskiego 37a, 60365 Poznan, Poland; 2 Department of Neurorehabilitation, Zürcher Reha Zentrum Wald, Faltigbergstrasse 7 8636 Wald, Switzerland

**Fig. 1:**
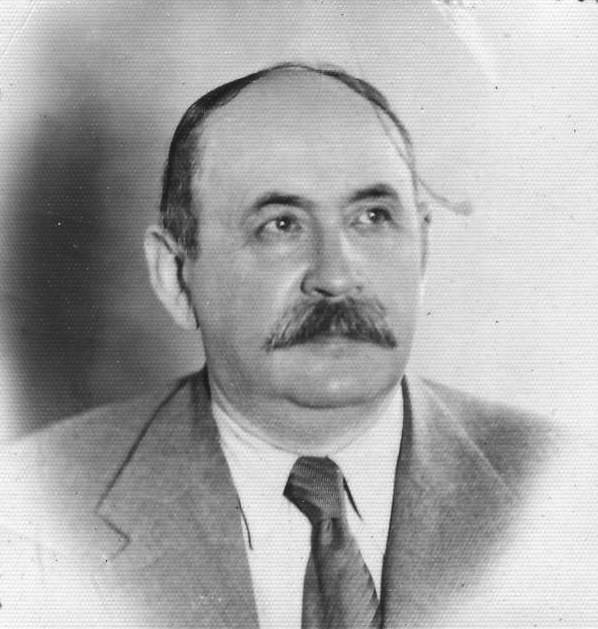
Władysław Sterling in the late 1930s, Personal Data Form (9 September 1940), the Chamber of Physicians (courtesy of Główna Biblioteka Lekarska im. Stanisława Konopki, Warsaw)

Władysław Sterling (Fig. 1) was born on January 14, 1877, in Warsaw in Congress Poland (an official part of the Russian Empire from 1867 to 1918). His parents and relatives were assimilated Jews, primarily artists, scholars, and physicians. It is no wonder that from his youth Sterling had a strong interest in both arts and medical science. He authored numerous essays, critiques, and poems, and more than 200 scientific papers in Polish, German, and French, including three previously undescribed neurological conditions. The best-known neurological eponyms associated with this distinguished Polish–Jewish medical figure being Flatau–Sterling syndrome and Sterling reflex.

After completing his secondary education in his native city, Sterling studied medicine at the Imperial University of Warsaw, graduating with distinction in 1900 and becoming a licensed physician in 1901. Afterwards, he was trained in the best neurological centres of the time in Western Europe. Consequently, he spent the following 3 years abroad, continuing his medical education at various renowned clinics in Germany and France. There, he carried out his first research in asthenic paralysis and psychology experiments in clinical settings [1]. His famous teachers included Herman Oppenheim (Berlin), Emil Kraepelin (Munich), and Józef Babiński (Paris).

After returning to Warsaw, Sterling was accepted for a position at the Neurology Department in the Jewish Orthodox Hospital, a healthcare institution with an excellent reputation located in the Czyste District. At that time, the Head of the Department was Professor Edward Flatau, an internationally respected scientist and neurologist, who founded the so-called Warsaw School of Neurology. The young physician quickly came to the attention of his superior and became one of his closest associates. In 1932, after Flatau’s death, Sterling took up his post and headed the Department with great enthusiasm and sacrifice until World War II broke out. In 1933, he defended his habilitation thesis at the University of Warsaw and was granted an exclusive right to lecture in neurology. Just 1 year later, he obtained veniam legendi in psychopathology and special needs education. During this time, he also conducted experimental and clinical research in addition to his work as a practicing physician and an academic teacher.

Sterling was also involved in numerous scientific societies and associations, for instance, the Polish Psychiatric Association (1920) and the Polish Psychological Association (1907). He was a founding member of the Warsaw Neurological Society, later transformed into the Polish Neurological Society (PNS), and in 1936 became its president. In 1910, together with Józef Babiński, Edward Flatau, Samuel Goldflam, Kazimierz Orzechowski and Jan Pilz, he inaugurated the journal *Neurologia Polska* [*Polish Neurology*]. In 1922, it became the official press organ of the PNS. Sterling was also one of the organizers of aided education in Poland and cooperated with the State Institute for Special Education established in 1922. Another eminent neurologist engaged in its activities was Józefa Joteyko, the most famous Polish woman scientist in Europe next to Marie Skłodowska-Curie [2].

After the Nazi occupation of Poland in September 1939, Warsaw’s Jewish Orthodox Hospital in Czyste was bombed and completely demolished. In the following months, stripped of his post, Sterling supported himself only by a private neurology and psychiatry practice. In November 1940, the Nazis sealed the Warsaw Ghetto. Sterling, along with thousands of other Polish Jews, was forced to move there. Shortly thereafter, he started to work at the neurology department in the Orthodox Jew Hospital relocated to the Ghetto. He also delivered a course of clandestine lectures in neurology to Jewish final year medical students and nurses. Less than 2 years later, in July 1942, the liquidation of the Warsaw Ghetto began. Sterling and his wife Róża, the sister of one of the most prominent serologists of the twentieth century Ludwik Hirschfeld, were smuggled out to the Aryan side of the Ghetto wall. Hidden from Germans in Nazi-occupied Warsaw, in conditions of maximum deprivation and defencelessness, constantly confronted with the threat of physical annihilation, they dedicated themselves solely to translating French poetry. After being denounced in the late 1943 or early 1944, they were shot down by murderers paid by the Gestapo [3].

Besides his outstanding contributions to psychiatry and special needs education, Władysław Sterling’s main field of activity was neurology. With Edward Flatau’s tradition behind him, and with his spirited commitment to clinical practice and scientific research, he had become one of the foremost representatives of neurology in pre-war Poland. In 1911, together with Flatau Sterling described one of the first cases of progressive torsion spasm (early onset torsion dystonia) in children, now known as Ziehen–Oppenheim syndrome [4]. Unlike Hermann Oppenheim’s and Theodor Ziehen’s papers, however, the study by the Warsaw scholars suggested that the disease had a genetic component [5]. Sterling’s name is also associated with dystrophia genito-sclerodermica [6], corticobasal degeneration (degeneration pyramido-extrapyramidalis) [7], and striatal and subcortical epilepsy (epilepsia extrapyramidalis) [8]. He also discovered and described in 1926 the Sterling reflex. It is a variant of Rossolimo’s finger sign, in which flexion of the fourth finger and the thumb is caused by a light stroke of the reflex hammer on the volar end of the two fingers [9]. Moreover, Sterling studied tumours of the nervous system, epidemic encephalomyelitis, hyperventilation-induced tetany, and various neuromuscular diseases [10]. He also noticed that innate predispositions for endocrine disorders affect the progression of epilepsy and other neurological conditions.

Like many other Jewish–Polish pioneers in neurology, Sterling died in the Holocaust. In his obituary published a few years after his tragic death Władysław Stein stated: “He was an extraordinary man, a neurologist with profound knowledge, a very talented clinician, and a physician of artistic calling” [2].
